# Generation and characterization of hepatocellular carcinoma cell lines with enhanced cancer stem cell potential

**DOI:** 10.1111/jcmm.13911

**Published:** 2018-10-02

**Authors:** Julienne K. Muenzner, Philipp Kunze, Pablo Lindner, Sandra Polaschek, Kira Menke, Markus Eckstein, Carol I. Geppert, Pithi Chanvorachote, Tobias Baeuerle, Arndt Hartmann, Regine Schneider‐Stock

**Affiliations:** ^1^ Experimental Tumor Pathology Institute of Pathology Friedrich‐Alexander University of Erlangen‐Nuremberg Erlangen Germany; ^2^ Institute of Pathology Friedrich‐Alexander University of Erlangen‐Nuremberg Erlangen Germany; ^3^ Department of Pharmacology and Physiology Faculty of Pharmaceutical Sciences Chulalongkorn University Bangkok Thailand; ^4^ Preclinical Imaging Platform Erlangen (PIPE) Institute of Radiology University Hospital Erlangen‐Nuremberg Erlangen Germany

**Keywords:** cancer stem cells, CD133, cell culture model, chorioallantoic membrane assay, hepatocellular carcinoma, tumour cell aggressiveness

## Abstract

Hepatocellular carcinoma (HCC) is one of the most common causes for cancer‐related death worldwide with rapidly increasing incidence and mortality rates. As for other types of cancers, also in HCC cancer stem cells (CSCs) are thought to be responsible for tumour initiation, progression and therapy failure. However, as rare subpopulations of tumour tissue, CSCs are difficult to isolate, thus making the development of suitable and reliable model systems necessary. In our study, we generated HepG2 subclones with enriched CSC potential by application of the spheroid formation method and subsequent single‐cell cloning. Analyses in several 2D and 3D cell culture systems as well as a panel of functional assays both in vitro and in vivo revealed that the generated subclones displayed characteristic and sustained features of tumour initiating cells as well as highly aggressive properties related to tumour progression and metastasis. These characteristics could clearly be correlated with the expression of CSC markers that might have prognostic value in the clinical HCC setting. Therefore, we conclude that our CSC enriched HepG2 clones certainly represent suitable model systems to study the role of CSCs during HCC initiation, progression and drug resistance.

## 1 INTRODUCTION

Hepatocellular carcinomas (HCCs) represent the most common type of liver cancer and one of the leading causes of cancer‐related deaths worldwide, having the most rapidly rising incidence and mortality rates when compared to other types of cancer.^1^, ^2^, ^3^, ^4^, ^5^, ^6^ In general, HCC tumours are genetically and phenotypically very heterogeneous as they arise from a background of different chronic pathological conditions such as liver cirrhosis and fibrosis that are most frequently associated with hepatitis B or hepatitis C virus infections, alcoholic liver diseases or metabolic disorders.^1^, ^7^, ^8^ Although there are various treatment strategies available, including conventional chemotherapy, surgical resection, liver transplantation and radiotherapy, the cure rate of HCC patients is very low.^1^, ^4^, ^9^, ^10^, ^11^ Besides the lack of highly reliable biomarkers to detect early‐stage tumours and the presence of primary liver diseases that limit the application of chemotherapeutics, the major reasons for poor patient outcome and high mortality rates associated with HCC are resistance to conventional anticancer therapy, a high frequency of recurrences and the formation of metastases.^1^, ^4^, ^8^, ^9^, ^10^, ^11^

As for other types of cancers also HCC tumours are thought to arise from so called cancer stem cells (CSCs) that represent a small sub‐population of tumour cells with stem cell‐like characteristics. These include unlimited self‐renewal capacity and the ability to differentiate into multiple tumour cell subtypes, which contributes to tumour heterogeneity.^1^, ^12^, ^13^, ^14^, ^15^, ^16^ In addition to tumour initiation and continuous tumour growth, hepatic cancer stem cells (HCSCs) are also thought to be responsible for tumour progression and metastatic spread. They are resistant to conventional chemo‐ or radiotherapy and remain in the healthy tissue after surgical resection of primary tumours, thus being majorly responsible for HCC recurrences.^1^, ^15^, ^16^, ^17^, ^18^ However, the specific roles of HCSCs and the mechanisms by which they act during individual stages of tumour growth and progression are still poorly understood. This hinders the development of efficacious HCSC‐targeting therapies for HCC patients that would eradicate CSC populations and promote a better patient outcome. To detect and specifically target certain HCSCs, it is first necessary to clearly identify highly aggressive CSC sub‐populations, analyse their cellular and signalling functions and finally elucidate their involvement in the individual processes of tumour initiation, progression and metastasis. Within the frame of current CSC research numerous markers including the cell surface proteins CD133 19, 20 and CD90,^21^ the detoxifying enzyme aldehyde dehydrogenase (ALDH)^22^ or the epithelial cell adhesion molecule (EpCAM)^23^, ^24^ have been identified and correlated with certain stem cell characteristics. Although distinct combinations of markers could be linked to specific aggressive HCSC sub‐populations using both in vitro and in vivo studies, so far it remains a major challenge to identify reliable CSC‐specific biomarker sets that can eventually be applied in a clinical setting to improve HCC diagnosis and treatment strategies.^1^, ^15^, ^16^, ^17^, ^18^

To better understand the role of CSCs during HCC initiation, growth, progression and drug resistance suitable and reliable model systems are required. These models will enable researchers to correlate certain CSC fractions to prognosis and to eventually develop novel HCSC biomarker sets as well as efficient CSC‐targeting treatment strategies. Mostly due to their ease of use and reasonable costs, cell culture based models represent the most frequent systems used to study CSCs and CSC associated features or mechanisms.^25^ In our study, we aimed to generate novel CSC enriched monoclonal cell lines of the well‐established HCC cell line HepG2. For this, we utilized the spheroid formation assay, which represents a commonly accepted method to enrich CSC populations.^25^, ^26^, ^27^ Our strategy gave rise to three distinct HepG2 sub‐cell lines, of which two HCSC enriched subclones were selected for detailed characterization. We could verify the suitability of our novel monoclonal sub‐cell lines as reliable, versatile and clinically relevant tools to investigate HCSC properties and aggressiveness by phenotypical and functional characterization both in vitro and in vivo in the chorioallantoic membrane (CAM) assay.

## 2 MATERIALS AND METHODS

Detailed information concerning the following methods can be found in the Supporting Information: Cell Lines and Culture Conditions, Morphology Analysis, Immunofluorescence Staining, Western Blot Analysis, RT‐qPCR for Stemness Markers, Tube Formation Assay, Histological Evaluation of CAM tumours and Metastasis Potential by Alu qPCR.

### 2.1 Generation of spheroid‐derived CSC enriched subclones

To generate CSC enriched sub‐cell lines of the established HCC cell line HepG2, we initially seeded HepG2 cells into Matrigel layers (40%‐50%, Corning^®^ Matrigel^®^ Growth Factor Reduced (GFR) Basement Membrane Matrix, Phenol Red‐Free, #356231) of a 6‐well cell culture plate, which were covered with cell culture medium (a total of three wells with 2 × 10^3^ single cells/well). After an incubation time of 10 days, in which the cell culture medium was exchanged every 2‐3 days, the spheroids formed by the HepG2 cells were harvested by trypsinization and single‐cell dilutions were prepared. As a single‐cell cloning step, 0.5 cells/well were then seeded into the wells of a 96‐well plate with pure cell culture medium. Clearly identified single‐cell clones that had formed three‐dimensional (3D) cell clusters after another 8 days of incubation were finally individually transferred into the wells of a 12‐well cell culture plate. Here, the growth of the spheroid‐like cell clusters continued for another 21 days, before they were harvested by trypsinization and single‐cell suspensions were prepared that were finally transferred to 6‐well cell culture plates. Further trypsinization steps to generate single‐cell suspensions were carried out to promote a two‐dimensional growth of the newly generated monoclonal spheroid‐derived and HCSC enriched HepG2 sub‐cell lines. Growth of the generated sub‐cell lines was monitored by light microscopy (Leica DMi1; Leica Microsystems, Wetzlar, Germany).

### 2.2 Spheroid migration/invasion assay

To assess the migratory potential of our HCSC enriched clones 3 and 5 in comparison to that of the parental HepG2 cells, we utilized an adapted spheroid migration assay protocol.^28^, ^29^, ^30^ For this, 1 × 10^3^ cells were seeded into the wells of a GravityPLUS™ Hanging Drop 96‐well plate (ISP‐06‐001, Perkin Elmer) and incubated for 4 days. For migration studies, spheroids were transferred to the wells of a 24‐well plate containing 500 μL of cell culture medium and incubated for an additional 4 days. Spheroid growth and tumour cell migration (with respect to the spheroid core size) were documented every 24 hours after transfer (0 hour) by light microscopy (Leica Dmi1 light microscope, 10× HI Plan I objective). Spheroid size and migration area were manually annotated using GIMP (GNU Image Manipulation Program, Version 2.8) and then finally determined in ImageJ 1.46r (Rasband, W.S., U.S. National Institutes of Health) applying a self‐written macro developed for this assay (*cf*. Supporting Information for details). The migration assay was carried out twice with at least three replicates per individual experiment and cell line depending on the spheroid formation capability. The migration assay was performed with the following total number of replicates: HepG2—n = 6 (3/3), K3: n = 9 (5/4), K5: n = 12 (7/5).

Spheroid invasion assays can be applied to analyse the invasiveness of cell lines in vitro.^30^, ^31^, ^32^ For this, multicellular spheroids (1 × 10^3^ cells/spheroid), generated with the GravityPLUS™ Hanging Drop system, were transferred to ultra‐low attachment round‐bottom 96‐well cell culture plates (#7007, Corning) and embedded in 200 μL of a 1:1 mixture of Matrigel and cell culture medium (Corning^®^ Matrigel^®^ Growth Factor Reduced (GFR) Basement Membrane Matrix, Phenol Red‐Free, #356231). After centrifugation (300 g, 3 minutes, 4°C), the plates were incubated at 37°C for 30 minutes and another 100 μL of cell culture medium were added onto the spheroid containing Matrigel layers. Spheroid invasion was documented by light microscopy every 48 hours for 8 days. Images were processed and analysed using ImageJ 1.46r (Rasband, W.S., U. S. National Institutes of Health, *cf*. Supporting Information for details). The spheroid invasion assay was carried out in two independent experiments with a total of 11 or 12 replicates per cell line (HepG2—n = 12 (6/6), K3: n = 11 (6/5), K5: n = 11 (6/5)).

### 2.3 Chorioallantoic membrane xenograft assay

The chorioallantoic membrane (CAM) assay represents a suitable and reliable alternative xenograft system to assess the in vivo growth and aggressiveness of tumour cells.^33^, ^34^ To analyse micro‐tumours formed by the HCSC enriched HepG2 subclones in comparison to those of their parental cell line, HepG2, clone 3 and clone 5 cells were grown on the CAM of fertilized chicken eggs. For this, specific pathogen‐free (SPF) eggs (VALO BioMedia, Osterholz‐Scharmbeck, Germany) were bred in an incubator at 37°C at a relative humidity of ~80%. On day 8 of chicken embryo development a window (Ø ~1‐1.5 cm) was cut into the more rounded pole of the eggs and the egg shell membrane was removed. The windows were re‐sealed with silk tape (Durapore^TM^, 3M) and the eggs were incubated for another day. Then 1 × 10^6^ cells were embedded in Matrigel (Corning^®^ Matrigel^®^ Basement Membrane Matrix, 356237; 1:1 mixture with medium; total volume 40 μL per pellet) and the resulting plugs were placed onto the CAM of the developing embryos (day 9 of incubation). Micro‐tumours with their surrounding CAM were harvested 5 days after engraftment (day 14 of embryonic development), imaged *ex ovo* and the tumour volume was determined as follows by assuming an ellipsoid shape: V_Tumour_ = length × width × height × 0.52.^35^ Finally, the CAM micro‐tumours were fixed in 4% phosphate buffered formalin for 24 hours, dehydrated and embedded in paraffin.

### 2.4 In vivo metastasis potential analysis by fluorescence imaging

To analyse the metastatic potential of clone five cells in comparison to the parental HepG2 cell line in vivo, the CAM assay was performed as described above, but using cells that were pre‐stained with a deep‐red live cell dye (Cell Proliferation Staining Reagent—Deep Red Fluorescence—Cytopainter; Abcam, Cambridge, UK, ab176736). Five days post‐engraftment of the cell pellets on the CAM, chicken embryos were removed from the eggs and decapitated. Embryos were then placed in an optical imaging system (IVIS Spectrum; Perkin Elmer, Waltham, MA, USA) and the optical signal of cells emitting the deep‐red fluorescence was acquired applying the following parameters: Epi‐illumination using an excitation filter of 605 nm and an emission filter of 660 nm, an exposure of 0.5 seconds and a field of view (FOV) of B: 6.6 cm. The average radiant efficiency within the embryos was determined by selecting a rectangular ROI that covered the entire embryo. Finally, the average radiant efficiency was corrected by the auto‐fluorescence signal of chicken embryos, where the CAM had been engrafted with unstained HepG2 cells.

### 2.5 Statistical analysis

All statistical analyses were performed with GraphPad Prism 7 (GraphPad Software, Inc., La Jolla, CA, USA).

## 3 RESULTS

### 3.1 HCSC enriched HepG2 subclones can be generated by spheroid formation and single‐cell cloning

**Figure 1:**
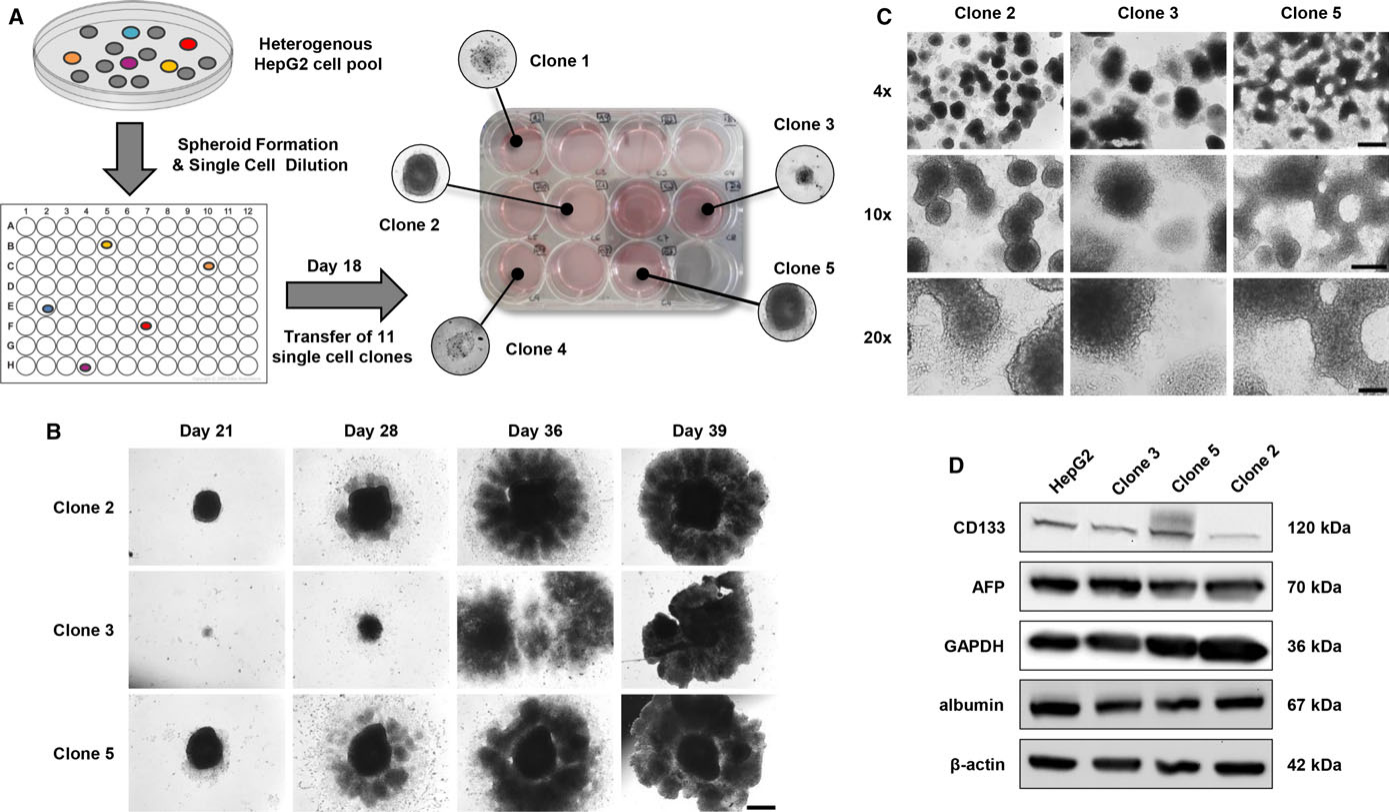
Generation of cancer stem cells enriched monoclonal HepG2 sub‐cell lines. A, Schematic overview of the generation of HepG2 sub‐cell lines by single‐cell cloning and spheroid formation. B, Spheroid growth of single‐cell clones 2, 3 and 5 (days 21‐39) after single‐cell cloning and transfer of generated spheroids from a 96‐well into a 12‐well cell culture plate on day 18 of incubation. Scale bar: 500 μm. C, 3D growth of clones 2, 3 and 5 (day 50) after trypsinization and transfer from a 12‐well into a 6‐well cell culture plate on day 39 of incubation. Scale bars: 4×—500 μm, 10×—250 μm, 20×—100 μm. D, Western Blot analysis of liver‐specific and hepatic cancer stem cells markers in clone 2, 3 and 5 cell populations in comparison to HepG2 cells. Western Blot images are representative for at least two independent experiments

To generate CSC enriched monoclonal sub‐cell lines of the well‐established and commonly used HCC cell line HepG2, we applied single‐cell cloning in combination with the spheroid formation strategy,^26^, ^27^ which represents a commonly applied and well‐accepted method to enrich CSC populations in tumour cell lines (Figure 1A). For this, we initially seeded single‐cell suspensions of HepG2 cells into the wells of a 6‐well cell culture plate containing a semi‐solid Matrigel matrix and harvested the herein formed and supposedly CSC enriched HepG2 spheroids after 10 days of incubation. By subsequent single‐cell cloning, we were able to generate eleven single‐cell clones (a total of 48 wells were seeded initially, ~23% of single‐cell clones) that were then transferred to a 12‐well cell culture plate (day 18). However, only five of the transferred clones actually adhered to the surface of the cell culture plate and finally only three single‐cell clones continued to grow as 3D spheroid‐like cell clusters, namely clone 2, clone 3 and clone 5 (Figure 1A). Noticeably, the formed spheroid‐like structures of all three clones remarkable increased in size within only 21 days of further incubation (Figure 1B). All three sub‐cell lines largely maintained their capability to grow in spheroid‐like and interconnected 3D structures even after harvesting by trypsinization and re‐seeding as single‐cell suspensions (Figure 1C). It should be mentioned, that this effect was most prominent for clone 5, which even formed network‐like structures. Only after several further cycles of trypsinization and re‐seeding of single‐cell suspensions all clones adapted to a mainly two‐dimensional (2D) growth pattern. We then started to analyse the expression of liver‐specific and HCSC markers in the 2D cultures of the three generated sub‐cell lines by Western Blot (Figure 1D) in comparison to the parental HepG2 cells. All spheroid‐derived HepG2 sub‐cell lines maintained their hepatocellular phenotype as verified by the detection of the liver‐specific markers α‐fetoprotein (AFP) and albumin, which are expressed at levels similar to those of the HepG2 cells. In contrast, clones 2, 3 and 5 exhibit a varied expression of the HCSC marker CD133. While the CD133 expression level in clone 3 was comparable to that of the parental HepG2 cells, this HCSC marker was strongly increased in clone 5, but apparently hardly expressed in clone 2. As CD133 represents one of the most commonly described HCSC markers that has been associated with self‐renewal capacity and tumorigenicity as well as drug‐resistance mechanisms, invasiveness, metastasis and poor prognosis,^17^, ^18^, ^19^, ^20^ we decided to focus on the two clearly CD133 positive clones 3 and 5. We verified the expression levels of CD133 and also evaluated the expression of two additional CSC markers (Figure S1), namely Nanog and Oct‐4, by qRT‐PCR. The mRNA expression of Nanog and Oct‐4 was increased in both clone 3 and clone 5, but a significantly elevated mRNA level could only be observed for Oct‐4 in clone 5 cells.

### 3.2 HCSC enriched clones show high cellular plasticity in vitro

**Figure 2:**
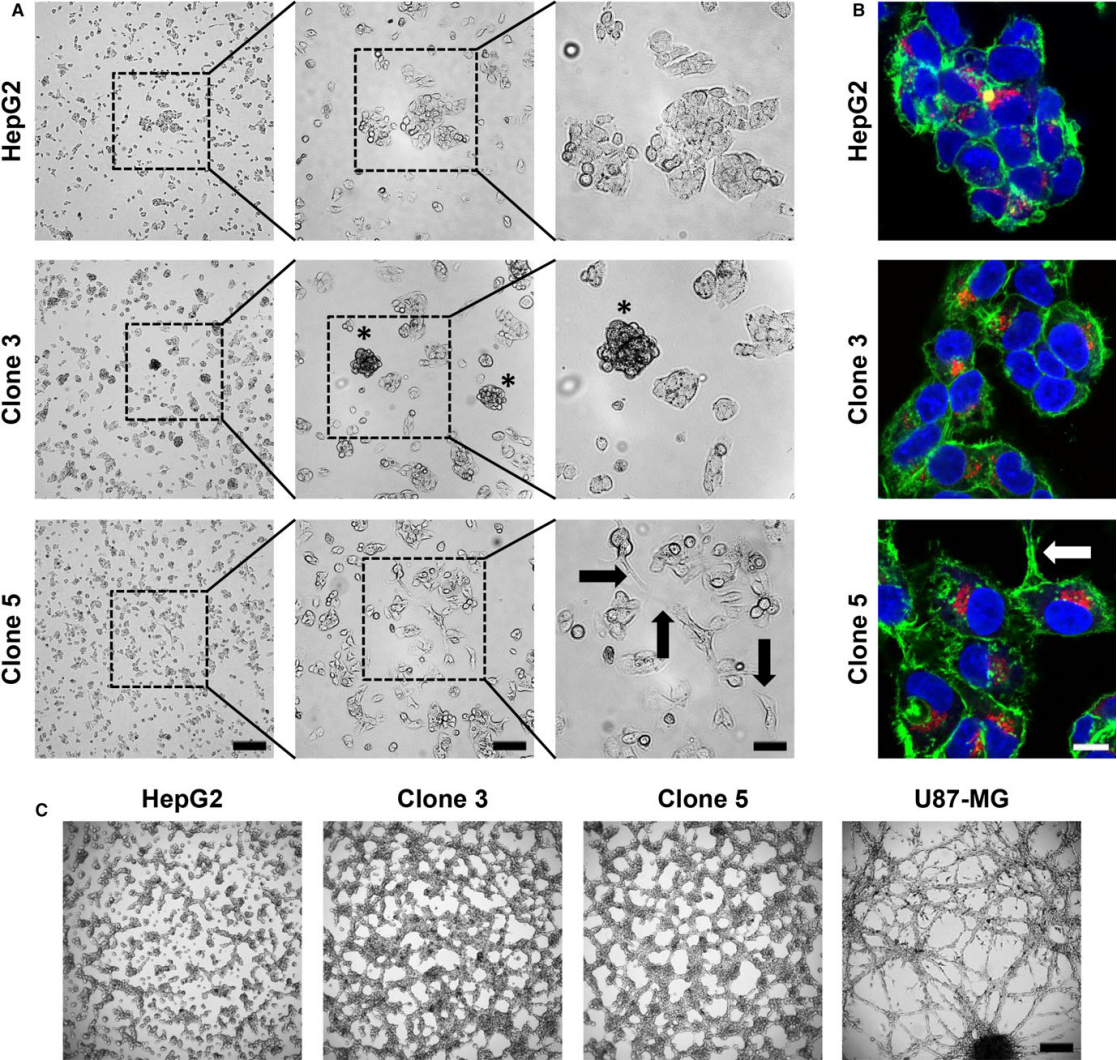
Cell morphology, growth pattern and tube formation ability of HepG2, clone 3 and clone 5 cells. A, Exemplary light microscopy images of HepG2, clone 3 and clone 5 cells cultured in 2D after 48 h of incubation (0 h—seeding of 1 × 10^6^ cells in a 10 cm culture dish). The experiment was carried out in triplicate. 4×—250 μm, 10×—100 μm, 20×—50 μm. Asterisks—spheroid‐like clusters; Arrows—elongated cell protrusions. B, Representative immunofluorescence staining images of HepG2, clone 3 and clone 5 cells. Green—F‐actin; red—AFP; blue—nuclei. Arrow—elongated cell protrusion. Scale—10 μm. C, Tube Formation assay to assess the ability of HepG2, clone 3 and clone 5 cells to form vasculogenic mimicry after growth on Matrigel for 24 h. Scale—250 μm. All images are representative for at least two independent experiments

Next, we investigated cell morphological features of the generated HCSC enriched cell lines by light microscopy and fluorescence staining (Figure 2A,B). While the parental HepG2 cells mainly grow as colonies in a monolayer, we could observe that both clone 3 and clone 5 cells have a higher tendency to form 3D structures. In this context, spheroid‐like clusters formed in cultures of both HepG2 subclones as exemplarily shown for clone 3 (asterisks in Figure 2A). In contrast to the parental HepG2 and clone 3 cells, which exhibited very similar rounded cell morphologies, clone 5 cultures showed large fractions of cells with a more bi‐ or tripolar morphology as well as elongated cell protrusions (arrows in Figure 2A,B). Fluorescence staining analysis revealed a more diffuse organization of the actin cytoskeleton and apparently an increase in the cytoplasmic/nuclear ratio for clone 3 and clone 5 when compared to HepG2 cells (Figure 2B). In addition, it should be noted that a similar expression level of the liver‐specific marker AFP could also be detected by immunofluorescence in all three investigated HepG2 cell lines corroborating our Western Blot data (Figures 1D and 2B). Since the aggressiveness of tumour cells with certain CSC phenotypes has largely been associated with high tumour cell plasticity such as the epithelial‐mesenchymal transition (EMT) and the formation of vasculogenic mimicry,^36^, ^37^, ^38^, ^39^ we subjected the HCSC enriched HepG2 clones 3 and 5 to a Matrigel‐based tube formation assay. Assessing their potential to generate 3D networks, we could indeed observe that the HCSC enriched clones display a much higher cell plasticity than the parental HepG2 cell line (Figure 2C). While HepG2 cells were only able to form very few aggregated tubule‐like 3D structures even at fairly high cell concentrations, both clone 3 and specifically clone 5 generated highly interconnected tubule‐like networks at much lower cell numbers (Figures 2C and S2) similar to those of the highly aggressive U87‐MG glioblastoma cell line, which was used as a positive control for vasculogenic mimicry formation.^40^, ^41^

### 3.3 HCSC enriched clones show a highly migratory and invasive potential in vitro

**Figure 3:**
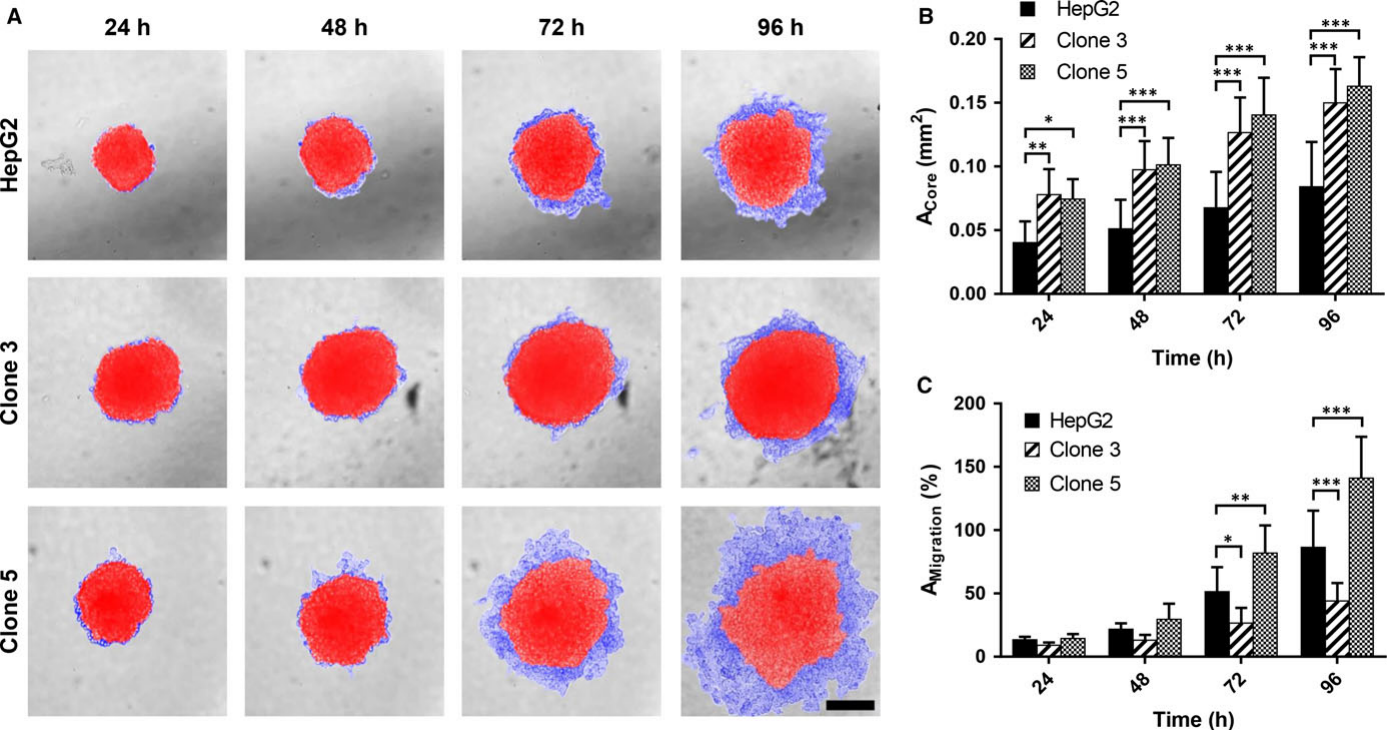
Spheroid growth and migratory potential of HepG2, clone 3 and clone 5 cells in vitro. A, Representative light microscopy images of the growth of HepG2, clone 3 and clone 5 spheroids and cell migration out of these spheroids after 24, 48, 72 and 96 h of incubation. Spheroid core area—red. Spheroid migration area—blue. Scale—200 μm. B, Time‐dependent spheroid growth as determined by measuring the spheroids’ core size after 24, 48, 72 and 96 h of incubation. C, Relative migration (A_M__igration_) of HepG2, clone 3 and clone 5 cells from spheroids as assessed by determination of the migration area with respect to the corresponding spheroid core area (A_C__ore_). Values represent means ± SD and statistical analysis was performed with two‐way ANOVA, followed by Dunnett's multiple comparisons test (HepG2: n = 6, clone 3: n = 9, clone 5: n = 12). *P < 0.05, **P < 0.01, ***P < 0.001

Tumour cell motility largely contributes to invasion and metastasis of tumours and can hence be applied as a measure for aggressiveness.^42^, ^43^ The migratory potential of the HCSC enriched clones 3 and 5 was assessed in a spheroid migration assay in comparison to HepG2 cells by determination of the migration area surrounding the spheroid (blue, Figure 3A) relative to the respective spheroid core size over time (red, Figure 3A). The spheroids of clones 3 and 5 were approximately twofold bigger in size (A_Core_) than those of the parental cell line throughout the whole experimental period (Figure 3B). In terms of migratory potential, clone 5 showed the highest cell motility, whereas the migratory potential of clone 3 was comparable or even a little lower than that of the parental HepG2 cells (Figure 3C).

**Figure 4:**
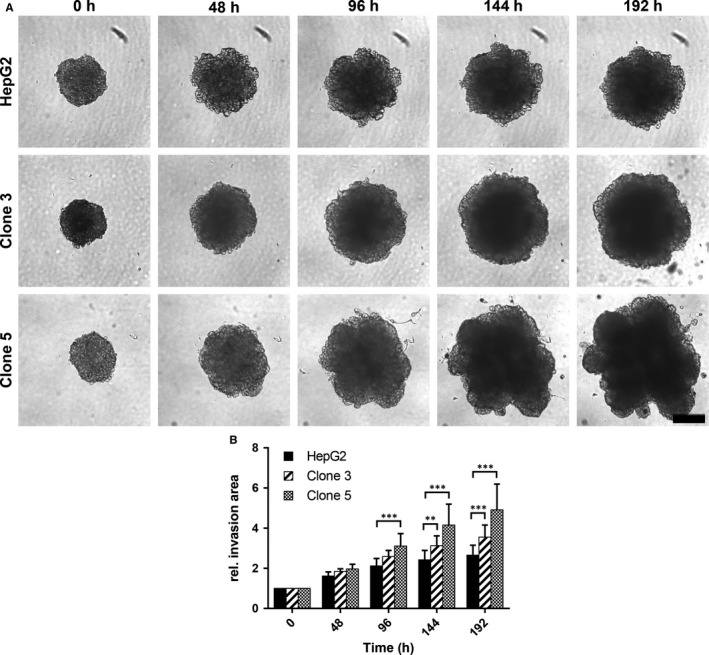
Invasiveness of HepG2, clone 3 and clone 5 cells in vitro. A, Representative light microscopy images documenting the invasive potential of HepG2, clone 3 and clone 5 cells after 48, 96, 144 and 192 h of incubation in the spheroid invasion assay (0 h—initial spheroids). Scale—200 μm. B, Time dependent relative invasion of HepG2, clone 3 and clone 5 cells as determined by measuring the area of the spheroids (size of initial spheroids at 0 h was set to 1). Values represent means ± SD and statistical analysis was performed with two‐way ANOVA followed by Dunnett's multiple comparisons test. (HepG2: n = 12, clone 3: n = 11, clone 5: n = 11). **P < 0.01, ***P < 0.001

To assess the invasive behavior of the cell lines in vitro, we performed a spheroid invasion assay, where spheroids of HepG2, clone 3 and clone 5 cells were embedded into Matrigel layers and invasion into this artificial extracellular matrix (ECM) was monitored over time (Figure 4A). While spheroids of the parental HepG2 cells only expanded by a factor of ~2.6 (192 hours) into the artificial ECM with respect to the initial spheroid size (size/rel. invasion area at 0 hour set to 1), a much higher invasion could be observed for clone 3 cells (rel. invasion area at 192 hours: ~3.6, Figure 4B). As in the spheroid migration assay clone 5 showed the most aggressive behavior and strongly invaded into the Matrigel layer reaching an approximately fivefold increased size after 192 hours of incubation (Figure 4B). In addition, it should be noted that highly irregular bleb‐like structures formed around the spheroids of clone 5, while the spheroids of the parental HepG2 and clone 3 cells largely maintained their regular sphere‐like shape. In general, this aggressive and invasive behavior of clone 5 in vitro can most likely be correlated to its phenotypical alterations with mesenchymal‐like features already described above (Figure 2).

### 3.4 HCSC enriched clones display pronounced tumour aggressiveness in vivo

**Figure 5:**
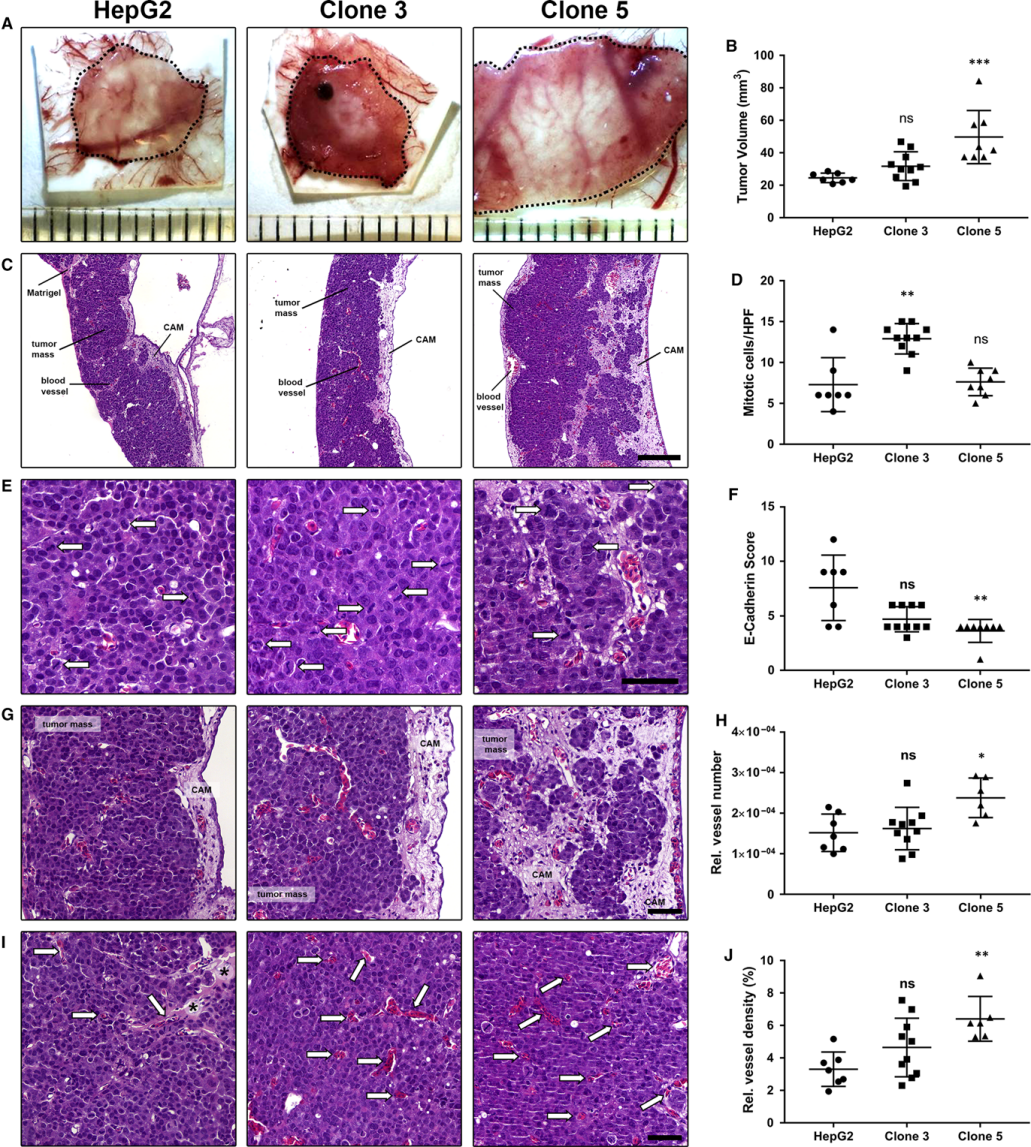
In vivo growth and aggressiveness of HepG2, clone 3 and clone 5 cells in the chorioallantoic membrane (CAM) xenograft assay. A, *Ex ovo* images of micro‐tumours harvested 5 days post‐engraftment on the CAM of fertilized chicken eggs. Dotted lines indicate tumour margins. Ruler segments define a length of 1 mm. B, Tumour volume of micro‐tumours after 5 days of incubation (HepG2: n = 7, clone 3: n = 10, clone 5: n = 8). C, Overview of HE stained micro‐tumour sections, Scale—200 μm. D, Proliferation of CAM‐micro‐tumours as determined by the average number of mitotic figures per HPF (HepG2: n = 7, clone 3: n = 10, clone 5: n = 8). E, Exemplary images of mitotic figures (arrows) as detected in HepG2, clone 3 and clone 5 micro‐tumours by HE staining. Scale—50 μm. F, E‐Cadherin immunoscore of HepG2, clone 3 and clone 5 micro‐tumours (HepG2: n = 7, clone 3: n = 10, clone 5: n = 8) as determined by immunohistochemical staining. G, Representative images of the invasion front in HE stained tissue section of HepG2, clone 3 and clone 5 CAM micro‐tumours. Scale—50 μm. H, Relative vessel number in CAM micro‐tumours formed by HepG2, clone 3 and clone 5 cells (HepG2: n = 7, clone 3: n = 10, clone 5: n = 6). I, Representative images of the vascularization in HepG2, clone 3 and clone 5 CAM micro‐tumours as seen in HE stained tissue sections. Scale—50 μm. Arrows—blood vessels filled with nucleated chick embryo erythrocytes. Asterisks—residual Matrigel. J, Relative vessel density of CAM micro‐tumours formed by HepG2, clone 3 and clone 5 cells (HepG2: n = 7, clone 3: n = 10, clone 5: n = 6). Data are presented with means ± SD and statistical analysis was performed using one‐way ANOVA, followed by Dunn's multiple comparisons test (Kruskal‐Wallis test). ns, not significant; *P < 0.05, **P < 0.01, ***P < 0.001

**Figure 6:**
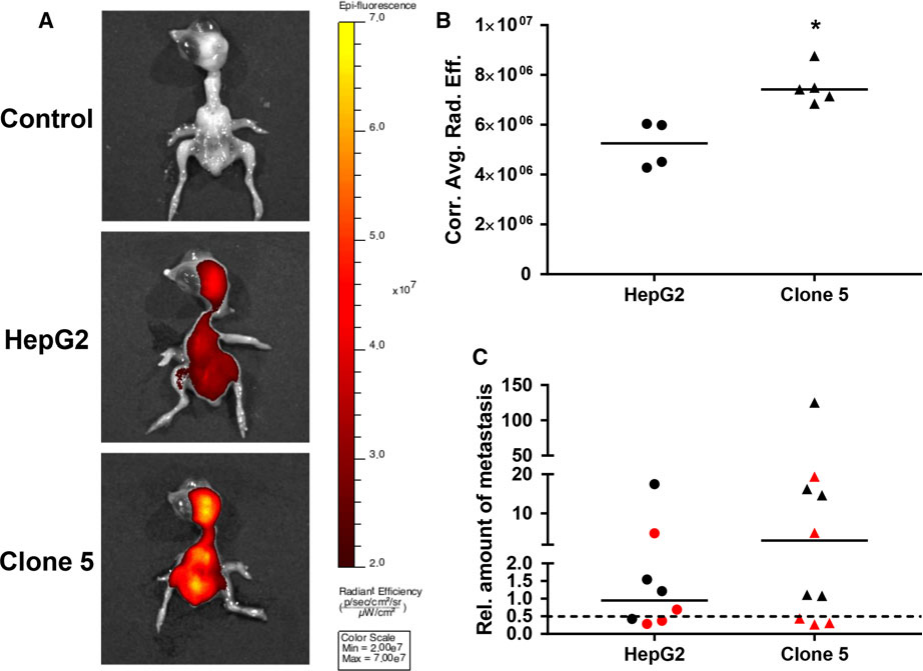
Fluorescence imaging of chicken embryos 5 days after engraftment of deep‐red fluorescence labelled HepG2 and clone 5 cell pellets to evaluate metastatic potential. A, Representative images of chicken embryos (top: control/background—n = 3; middle: HepG2—n = 4; bottom: clone 5—n = 5) with colour‐coded overlays of the radiant efficiency as determined by fluorescence imaging with a range from black to yellow according to the given colour bar (IVIS Spectrum, Perkin Elmer). B, Quantified average radiant efficiencies as detected by fluorescence imaging in chicken embryos after engraftment of parental HepG2 (n = 4) and clone 5 (n = 5) cells. C, Relative amounts of metastasis in the liver (black) and brain (red) of chicken embryos as determined by Alu real‐time PCR 5 days after engraftment of the HepG2 and clone 5 cells. A value of 1 indicates an amount of 0.01 ng of human DNA. The dotted line represents the cut‐off for metastasis detection, which was defined by the sensitivity limit of the Alu PCR method. Medians of the data are presented as lines in the graphs and statistical analysis was performed with the Mann‐Whitney test. *P < 0.05.

To confirm the more aggressive stem‐like and invasive phenotype of the spheroid‐derived clones 3 and 5 in comparison to the parental HepG2 cells and to evaluate their tumour forming capacity in vivo, the chicken CAM xenograft assay was applied (Figure 5). Here, the HCSC enriched clones 3 and 5 did not only establish larger tumours than the parental HepG2 cell line (HepG2: n = 7; clone 3: n = 10; clone 5: n = 8), but tumours formed by clones 3 and 5 also showed further characteristics of aggressive growth, which could already be observed by simple macroscopic and microscopic investigation of the CAM micro‐tumours (Figure 5A‐C). Representative overview images of the different growth patterns of tumours formed by parental HepG2, clone 3 and clone 5 cells are shown in Figure 5C. When evaluating the HE stained tumour sections in more detail, we found a significantly enhanced proliferation, as determined by the mitotic rate of cells, only for clone 3 cells at day 5 post‐engraftment (Figure 5D,E). In accordance with the highly invasive potential of clone 5 observed in vitro (Figure 4), clone 5 CAM micro‐tumours showed a loss of E‐cadherin (Figure 5F), a highly infiltrative growth at the tumour invasive front as well as a considerable interaction/mixing with the CAM tissue throughout the whole tumour mass (Figure 5G). The decrease in E‐Cadherin expression in clone 5 cells and an absence of Vimentin in all investigated HepG2 tumours (in vivo data for Vimentin not shown) were additionally verified in vitro by Western Blot (Figure S3). The reduced E‐Cadherin expression and the presence of loosely packed tumour masses in clone 5 micro‐tumours seem to indicate a loss of cell adhesion that could be related to the process of EMT and the promotion of tumour cell metastasis.^25^, ^44^ In addition, also the significantly higher vascularization of clone 5 micro‐tumours (Figure 5H‐J) represents an essential hallmark of cancer that might be associated with metastatic spread.^45^ When investigating the metastatic potential of the highly aggressive HCSC enriched clone 5 by fluorescence imaging 5 days post‐engraftment of the micro‐tumours (Figure 6A), we indeed observed a significantly increased metastatic spread of clone 5 cells in chicken embryos (Figure 6B). The enhanced metastatic potential of clone 5 cells was further verified by detection of human DNA in selected embryo organs using an Alu‐specific qPCR (Figure 6C).

## 4 DISCUSSION

Hepatic cancer stem cells are considered as the tumour initiating and heterogeneity promoting HCC cell populations.^1^, ^12^, ^15^, ^16^, ^17^, ^18^ Besides their role in tumour formation and malignant tissue maintenance, HCSCs have also been reported to be involved in tumour progression, metastatis related processes and drug resistances, thereby largely contributing to HCC recurrences and therapy failure.^1^, ^12^, ^15^, ^16^, ^17^, ^18^ Hence, the investigation of HCSCs and detailed elucidation of their role in tumour initiation, progression, metastasis and drug resistance has become a major focus in HCC research.^1^, ^13^, ^15^, ^16^, ^17^, ^18^ As rare subpopulations of tumour tissue CSCs are difficult to isolate and study, so that the development of suitable and reliable cell culture model systems is required.^25^ These models can then be used to better understand the role of HCSCs during carcinogenesis and tumour progression. In turn, this improved understanding will eventually allow us to identify novel treatment strategies that efficiently target highly aggressive and resistant HCSC subpopulations being responsible for therapy failure.^25^

In our present study, we successfully generated two HepG2 derived monoclonal tumour cell lines, namely clone 3 and clone 5, with enhanced CSC potential by application of the tumour sphere formation assay, which represents a commonly accepted method to enrich CSC populations,^25^, ^26^, ^27^ and subsequent single‐cell cloning. The established clones 3 and 5 were positive for the cell surface protein CD133, which is one of the most commonly described HCSC markers. It is associated with self‐renewal capacity, tumorigenicity, chemoresistance, invasiveness, tumour angiogenesis and metastasis.^16^, ^17^, ^18^, ^19^, ^20^, ^46^ In addition, a higher expression of the CSC markers Nanog and Oct‐4 could be observed, which are known to be preferentially co‐expressed in CD133^+^ HCSCs and to be involved in the induction of EMT.^1^, ^17^, ^47^, ^48^ To confirm the stemness state and analyse the potentially correlated tumour cell aggressiveness of the spheroid‐derived HepG2 clones 3 and 5, several functional assays were performed in vitro and in vivo. Especially, clone 5 was proven to exhibit characteristics associated with a highly aggressive and invasive phenotype, most likely due to its significantly higher expression in clinically relevant stemmness markers and more mesenchymal‐like features. In 2D culture both clone 3 and clone 5 showed a higher tendency to build up and grow in 3D structures in comparison to the parental HepG2 cell line. In addition, enhanced cell plasticity in the tube formation assay, which is well known of being associated with EMT and the CSC phenotype as well as invasion and metastasis,^36^, ^37^, ^38^, ^39^, ^49^, ^50^ could be detected for the spheroid‐derived clones when grown on an artificial ECM. Intriguingly, also a significantly greater migratory and invasive potential was observed for clone 5 in vitro. Moreover, we showed that the CAM assay does not only represent a suitable alternative xenograft system to evaluate tumorigenicity, but can also be applied to study various hallmarks of cancer^45^ such as proliferation, invasion, angiogenesis and metastatic potential of tumour cells.^33^, ^34^, ^51^ Here, clone 5 displayed highly aggressive properties as well, forming the largest CAM micro‐tumours with very strong vascularization, haemorrhagic areas and a highly infiltrative growth as observed by considerable mixing of cell clusters with the CAM tissue. In addition, a greater metastatic potential could be detected for clone 5 cells when compared to the parental HepG2 cell line in vivo by fluorescence imaging and human Alu‐specific qPCR. These observations might also explain the lower mitotic rate identified in clone 5 micro‐tumours, even though these were the largest tumours formed. The strong mixing of CAM tissue with clone 5 tumour masses results per se in less tumour cells per evaluated HPF when compared to the CAM tumours formed by clone 3 or the parental HepG2 cells. In addition, it is well known that the process of EMT and hence the dissemination of tumor cells to form distant metastases are dependent on a reduced proliferation rate.^25^, ^44^ Taken all together and in accordance with literature,^44^, ^52^, ^53^, ^54^ our data indicate that the combination of an enhanced CSC potential and EMT(‐like) features that contribute to migration and invasion also seems to be largely responsible for a highly aggressive and metastatic phenotype in our HCSC model system both in vitro and in vivo.

In conclusion, we successfully demonstrated that characteristic features of tumour initiating cells are clearly correlated with highly aggressive tumour cell properties in our cell culture model, which in turn can be linked to tumour progression and metastasis in the clinical HCC setting. Thus, we suggest that the establishment of HCC cell lines with a sustained enriched CSC potential by tumour sphere formation and single‐cell cloning represents a suitable and simple approach to develop reliable cell culture model systems for the investigation of HCSCs and associated tumour aggressiveness.

## CONFLICT OF INTEREST

The authors confirm that there are no conflicts of interest.
