# Supplementary material for: Generation and characterization of hepatocellular carcinoma cell lines with enhanced cancer stem cell potential
Source: J Cell Mol Med. 2018 Oct 2;22(12):6238–48. doi: 10.1111/jcmm.13911 (PMC6237557; doi:10.1111/jcmm.13911)
Supplement: Supplementary file 1 [file JCMM-22-6238-s001.doc]

**Supporting Information**

**Generation and characterization of hepatocellular carcinoma cell lines with enhanced cancer stem cell potential**

Julienne K. Muenzner,^1,2^ Philipp Kunze,^1,2^ Pablo Lindner,^1,2^ Sandra Polaschek,^1,2^ Kira Menke,^1,2^ Markus Eckstein,^2^ Carol I. Geppert,^2^ Pithi Chanvorachote,^3^ Tobias Bäuerle,^4^ Arndt Hartmann,^2^ Regine Schneider-Stock^1,2^*

^1^Experimental Tumor Pathology, Institute of Pathology, Friedrich-Alexander University of Erlangen-Nuremberg, Erlangen, Germany

^2^Institute of Pathology, Friedrich-Alexander University of Erlangen-Nuremberg, Erlangen, Germany

^3^Department of Pharmacology and Physiology, Faculty of Pharmaceutical Sciences, Chulalongkorn University, Bangkok, Thailand

^4^Preclinical Imaging Platform Erlangen (PIPE), Institute of Radiology, University Hospital Erlangen-Nuremberg, Erlangen, Germany

**Table of Content**

| ***Methods*** | **S1-S7** |
| --- | --- |
| *Cell Lines and Culture Conditions* | S1 |
| *Western Blot* | S1-S2 |
| *RT-qPCR for stemness markers* | S2-S3 |
| *Morphology Analysis* | S3 |
| *Immunofluorescence Staining* | S3 |
| *Tube Formation Assay* | S4 |
| *Spheroid Migration Assay – ImageJ Macro* | S4 |
| *Spheroid Invasion Assay – ImageJ Macro* | S5 |
| *Histological Evaluation of CAM micro-tumors* | S5-S6 |
| *Metastasis Potential by Alu qPCR* | S6-S7 |
| ***Results*** | **S7-S8** |
| *HCSC enriched HepG2 clones show an increased expression of CSC markers* | S7 |
| *Tube Formation Assay - Results* | S8 |
| *E-Cadherin expression is strongly reduces in clone 5 cells* | S8 |
| ***References*** | **S9** |

**Methods**

*Cell Lines and Culture Conditions*

The human hepatocellular carcinoma cell line HepG2 was obtained from the ATCC (HB-8065) and kept in Dubelco’s modified Eagle’s medium (DMEM, 41965039, Gibco/Life Technologies) supplemented with 10% FBS (P30-3306, PAN Biotech) and 1% Penicillin/Streptomycin (P06-07100, PAN Biotech). Cells were cultured in a humidified atmosphere at 37 °C with 5% CO_2_. All generated HepG2 subclones and the U87-MG glioblastoma cell line, which was a kind gift from Prof. Dr. Dr. C. Mawrin, Department for Neuropathology, University Clinic Magdeburg, were cultured under identical conditions. The HCT116 p21-/- cell line was kindly provided by Bert Vogelstein (Johns Hopkins) and kept in DMEM medium (41965039, Gibco/Life Technologies) supplemented with 10% FBS (P30-3306, PAN Biotech), 1% Penicillin/Streptomycin (P06-07100, PAN Biotech), 1% sodium pyruvate solution (S8638, Sigma-Aldrich) and 1% MEM-Non-Essential Amino Acids Solution (11140-035, Gibco), but otherwise cultured under identical conditions. Cell lines were authenticated using Multiplex Cell Authentication by Multiplexion (Heidelberg, Germany) as described by Castro *et al.* [S1] . Mycoplasma free status has been verified for all cell lines.

*Western Blot*

Cell pellets were collected from cell cultures with ~80% confluency and lysed in Urea buffer (4 M Urea, 0.5% SDS, 62.5 mM Tris, pH 6.8) supplemented with 1% PMSF (Carl Roth GmbH) and 1% PIC (Merck Millipore) on ice for 1 h (samples were vortexed every 10 min). Finally, lysates were sonicated, centrifuged (14.000 rpm, 10 min, 4 °C) and the protein concentration was determined using the DC^TM^ Protein Assay Kit (Bio-Rad) and a VICTOR Multilabel Plate Reader (Perkin Elmer) according to the manufacturer’s instructions. Equal amounts of protein (40 µg or 60 μg for all HepG2 cell lines, 30 µg for HCT-116 p21 -/- cells) were separated by denaturing SDS-PAGE, transferred onto nitrocellulose membrane overnight using a Mini-PROTEAN® Tetra Vertical Cell Systems (Bio-Rad). Protein bands were subsequently visualized using Immobilon Western Blot Chemiluminescent HRP Substrate (Merck Millipore) and the antibodies listed below. Signal detection was performed using a GeneGnome (Syngene) Western Blot detection system and GAPDH (1:50000, GAPDH monoclonal antibody, clone 6C5 (HRP), MAB5476, Abnova) or β-actin (1:10000, monoclonal anti-β-actin, clone AC-7, A2228, Sigma Aldrich) were applied as loading controls. Primary antibodies used: CD133 (1:250, CD133/1 (W6B3C1) pure, human, 130-092-395, Miltenyi Biotec), AFP (1:2000, AFP (3H8) mouse mAb, #3903, Cell Signaling), albumin (1:1000, albumin antibody, 4929, Cell Signaling), E-Cadherin (1:1000, E-Cadherin (24E10) Rabbit mAb, #3195, Cell Signaling), Vimentin (1:5000, Vimentin (D21H3) XP® Rabbit mAb, #5741, Cell Signaling). Secondary antibodies used: goat anti-rabbit IgG (H+L) secondary antibody, HRP (1:10000, 31460, Thermo Fisher Scientific) and goat anti-mouse IgG (H+L) secondary antibody, HRP (1:10000, 31460, Thermo Fisher Scientific). Band intensities were determined in ImageJ 1.46r (Rasband, W.S., U.S. National Institutes of Health).

*RT-qPCR for stemness markers*

For RT-qPCR analysis RNA was isolated from HepG2, clone 3 and clone 5 cell pellets using the QIAzol® Lysis Reagent (Qiagen) and the RNeasy Mini Kit (Qiagen). RNA concentration was determined with a Nanodrop® ND-1000 system (Nanodrop) and reverse transcription was performed using the QuantiTect Reverse Transcription Kit (Qiagen). Amplification of cDNA was then achieved by application of gene-specific primers (Metabion) and using the QuantiTect SYBR® Green PCR Kits (Qiagen) according to the manufacturer’s instructions. Primers applied in RT-qPCR experiments are given in the following Table S1. Measurement of expression values was performed using a CFX96^TM^ Real-Time System (Bio-Rad) and the C1000^TM^ Thermal Cycler (Bio-Rad). Expression values were normalized to human B2M expression. Finally, mRNA expression levels were determined relative to HepG2 control samples (set to 1). The experiment was performed in biological triplicate with technical quadruplicates each.

**Table S1:** Primers used in RT-qPCR experiments (Metabion).

| **Primer** | **Sequence** |
| --- | --- |
|  |  |
| B2M.FW | 5'-AGCAGCATCATGGAGGTTTG-3' |
| B2M.RV | 5'-AGCCCTCCTAGAGCTACCTG-3' |
| CD133.FW | 5’-ATCCACAGATGCTCCTAAGGCT-3’ |
| CD133.RV | 5’-CTCCCCGACAGTGCGATG-3’ |
| NANOG.FW | 5’-AATACCTCAGCCTCCAGCAGATG-3’ |
| NANOG.RV | 5’-TGCGTCACACCATTGCTATTCTTC-3’ |
| OCT-4.FW | 5’-GAGAACCGAGTGAGAGGCAACC-3’ |
| OCT-4.RV | 5’-CATAGTCGCTGCTTGATCGCTTG-3’ |

*Morphology Analysis*

To investigate the morphology of the HCSC enriched subclones 3 and 5 in comparison to HepG2 cells, an equal number of 1 × 10^6^ cells was seeded into 10 cm cell culture dishes. After 48 h of incubation light microscopy images were taken using a Leica DMi1 light microscope (inverse, 4x, 10x, 20x objective HI Plan I, Leica Microsystems).

*Immunofluorescence Staining*

HepG2, clone 3 and clone 5 cells (2 × 10^5^ cells/well) were seeded onto coverslips (Ø 12 mm, Thermanox® Plastic Coverslips, Nunc) in 12-well cell culture plates. After 24 h of incubation, cells were fixed with 4% phosphate-buffered formalin (20 min), blocked and permeabilized in a 0.1% Triton X-100/1% BSA solution (in PBS) for 30 min, and finally stained with specific antibodies and fluorescent conjugates/dyes as indicated below. Anti-α-fetoprotein was visualized using the AFP (3H8) mouse monoclonal antibody (1:200 dilution in 1% BSA, 1 h at 37 °C, #3903) and an AlexaFluor® 555 conjugated anti-mouse IgG antibody (1:500 dilution in 1%BSA, 1 h at room temperature, #4409) from Cell Signaling. F-actin was stained by AlexaFluor® 488 conjugated Phalloidin (1:100 in PBS, #8878, Cell Signaling) and nuclei by DAPI, which was present in the ProLong^TM^ Gold Antifade mountant used (P36931, Thermo Fisher Scientific).

*Tube Formation Assay*

The ability of the HCSC enriched HepG2 sub-cell lines to build up organized and interconnected 3D networks of tubule-like structures was assessed by performing the Tube Formation Assay [S2, S3] . For this, HepG2, clone 3 and clone 5 cells (3 × 10^4^ , 4 × 10^4^  and 5 × 10^4^ cells/well) were seeded on growth factor reduced Matrigel (Corning® Matrigel® Growth Factor Reduced (GFR) Basement Membrane Matrix, Phenol Red-Free, #356231) in the wells of a 96-well cell culture plate, incubated for 24 h and generated networks were then documented using a Leica DMi1 light microscope with a 4x objective (HI Plan I, Leica Microsystems). The highly aggressive U87-MG glioblastoma cell line was used as a positive control (3 × 10^4^ and 4 × 10^4^ cells/well) [S2, S3] .

*Spheroid Migration Assay – ImageJ Macro*

run("Channels Tool... ");

run("Make Composite");

Stack.setDisplayMode("grayscale");

Stack.setChannel(2);

setAutoThreshold("Default dark");

//run("Threshold...");

setThreshold(0, 90);

run("Convert to Mask", " ");

Stack.setChannel(3);

//run("Threshold...");

setAutoThreshold("Default dark");

setThreshold(180, 255);

run("Convert to Mask", " ");

Stack.setChannel(2);

run("Analyze Particles...", "size=100-Infinity pixel circularity=0.00-1.00 show=Nothing display slice");

Stack.setChannel(3);

run("Analyze Particles...", "size=100-Infinity pixel circularity=0.00-1.00 show=Nothing display slice");

*Spheroid Invasion Assay – ImageJ Macro*

run("Channels Tool... ");

run("Make Composite");

Stack.setDisplayMode("grayscale");

Stack.setChannel(3);

//run("Threshold...");

setAutoThreshold("Default dark");

setThreshold(180, 255);

run("Convert to Mask", " ");

Stack.setChannel(3);

run("Analyze Particles...", "size=100-Infinity pixel circularity=0.00-1.00 show=Nothing display slice");

*Histological Evaluation of CAM micro-tumors*

Serial sections (2 µm) were cut from paraffin blocks and mounted on pre-coated slides to perform histological evaluations of the CAM tumors. Sections were stained with hematoxylin and eosin according to standard procedures for histomorphological analyses. All stained HE slides were scanned using a Panoramic MIDI system (Camera type: CIS VCC-FC60FR19CL, objective: Plan-Apochromat, magnification: 40x, Camera adapter magnification: 1x) (3DHISTECH) for digital analysis. Adjacent slides were stained with specific antibodies against E-Cadherin (1:2000, clone 36/E-Cadherin, BD Biosciences) and Vimentin (1:200, clone V9, Dako) according to standard routine procedures for immunohistochemical analysis. The E-Cadherin immunoreactive score (IRS) was determined by multiplying the staining intensity (0-3) with the respective level of positive cells (0-4) according to Remmele and Stegner [S4, S5] . The levels of positive cells are defined as follows: no positive cells - 0, < 10% positive cells – 1, 10% to 50% positive cells – 2, 51% to 80% positive cells – 3, and > 80% positive cells – 4. The mitotic rate of cells in the CAM micro-tumors was determined using HE stained sections and the high-power-field (HPF, 40x) method (HepG2: n = 7; clone 3: n = 10; clone 5: n = 8). The mitotic rate was then calculated as the mean number of mitotic figures in five HPFs of each sample. However, in two micro-tumors of the HepG2 parental cell line there were only 2 and 3 HPFs present that could be used for proper analysis of the mitotic rate. The vessel density of the CAM micro-tumors was assessed by analysis of the same HE stained sections applying the Case Viewer software (Version 2.0, 3DHISTECH). To determine the tumor area covered by blood vessels, only intratumoral vessels that contained nucleated chicken erythrocytes were considered (HepG2: n = 7; clone 3: n = 10; clone 5: n = 8). The relative vessel density (intratumoral) was then calculated by dividing the area covered with blood vessels by the total micro-tumor area. Large intratumoral white spaces or larger areas without focus were present in a few cases of the scans due to cutting artefacts and were excluded from the analysis.

*Metastasis Potential by Alu qPCR*

After determination of the *in vivo* metastasis potential in the CAM assay by fluorescence imaging selected chicken embryo organs (liver and brain) were harvested and stored at -80°C until further use for metastasis detection by Alu qPCR. Dissociation of chicken embryo organs was performed using the gentleMACS^TM^ Octo Dissociator system with M tubes (Miltenyi Biotec) according to the supplier’s protocol. Optimized cell lysis (16 h, 56 °C) and subsequent genomic DNA preparation were performed by application of the NucleoSpin® Tissue Kit (Macherey-Nagel) according to the user manual. Isolated DNA was quantified and checked for purity using a Nanodrop® ND-1000 system (Nanodrop). Amplification of human Alu sequences (hAlu) in the DNA samples (200 ng of total genomic DNA) was then achieved by application of specific primers [6] (Table S2, Metabion) and the QuantiTect SYBR® Green PCR Kit (Qiagen) according to the manufacturer’s instructions. Quantification of the human Alu sequence content was performed using a CFX96^TM^ Real-Time System (Bio-Rad) and the C1000^TM^ Thermal Cycler (Bio-Rad). Samples were subjected to an initial denaturation of 95°C for 15 min, followed by 40 cycles at 94°C for 15 s (denaturation), 60°C for 30 s (annealing), and 72°C for 30 s (elongation). Values were normalized to chicken GAPDH [S6] (chGAPDH, specific primers in Table S2, Metabion) and relative hAlu sequence levels (relative amount of metastasis) were determined with respect to a human genomic DNA control sample (Human Genomic DNA, Human Mixed, 100 µg, G3041, Promega). A human genomic DNA amount of 0.01 ng was defined as a relative value of 1.0. The sensitivity limit of the Alu PCR method could be detected as 0.001 ng of human genomic control DNA samples reached relative values of ~0.3, even though a value of 0.1 would be expected. For this, we decided to define strict cut-off for metastasis detection in this specific PCR method, thus relative values < 0.5 were defined as no metastasis present. The experiment was performed in technical triplicates per sample (HepG2: n = 4 and clone 5: n = 5).

**Table S2:** Primers used for metastasis detection in chicken embryo organs [6] (Metabion).

| **Primer** | **Sequence** |
| --- | --- |
|  |  |
| hAlu.sense | 5'-ACGCCTGTAATCCCAGCACTT-3' |
| hAlu.antisense | 5'-TCGCCCAGGCTGGAGTGCA-3' |
| chGAPDH.sense | 5'-GAGGAAAGGTCGCCTGGTGGATCG-3' |
| chGAPDH.antisense | 5'-GGTGAGGACAAGCAGTGAGGAACG-3' |

**Results**

*HCSC enriched HepG2 clones show an increased expression of CSC markers*


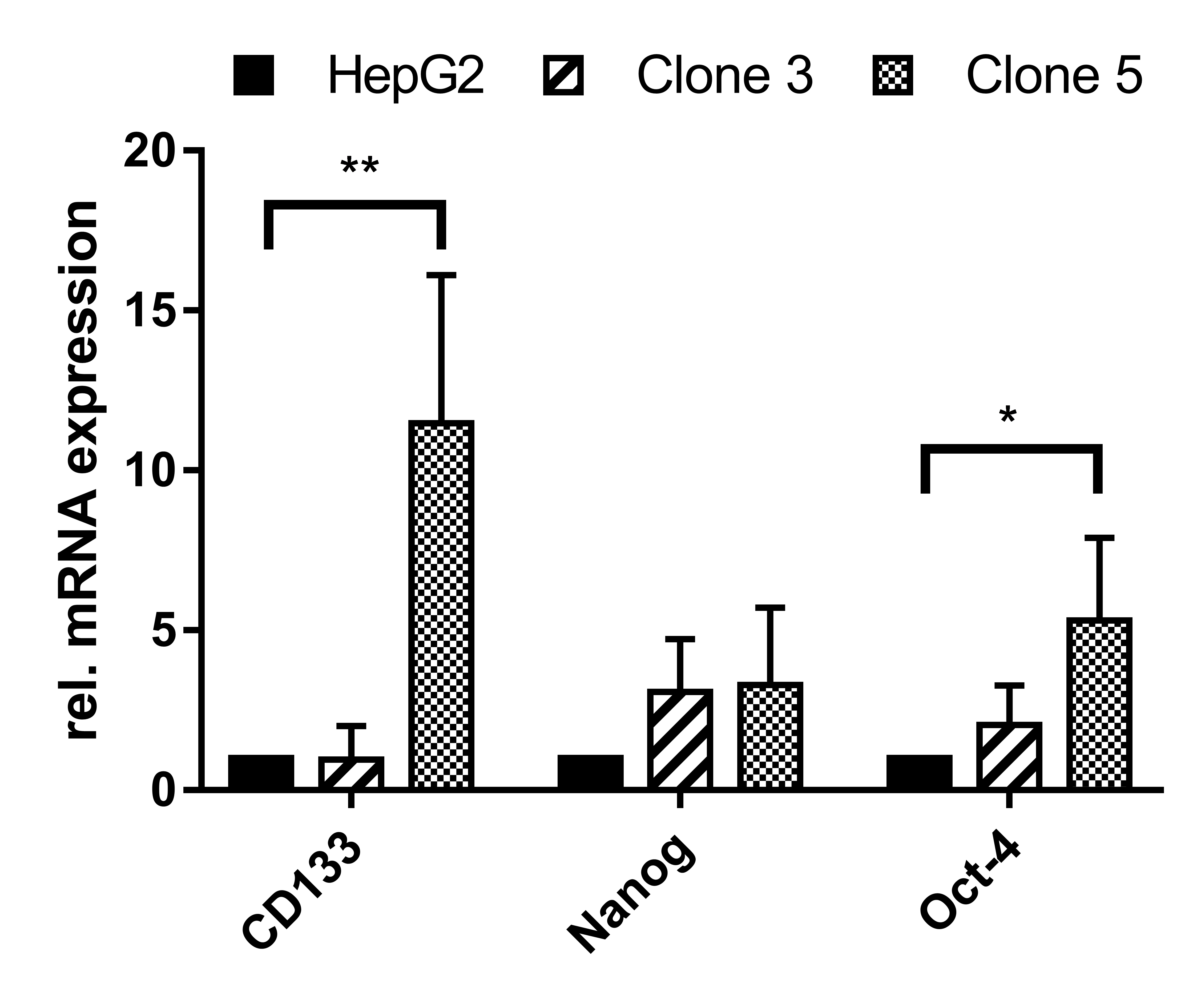


**Figure S1:** Relative mRNA expression of the stemness markers CD133, Nanog and Oct-4 in parental HepG2 (rel. expression set to 1 for each marker), clone 3 and clone 5 cells (for all cell lines n = 3). Values represent means ± SD and statistical analysis was performed using one-way ANOVA followed by Dunnett’s multiple comparisons test. * indicates p ≤ 0.05, ** indicates p ≤ 0.01.

*HCSC enriched HepG2 clones show a higher tumor cell plasticity*


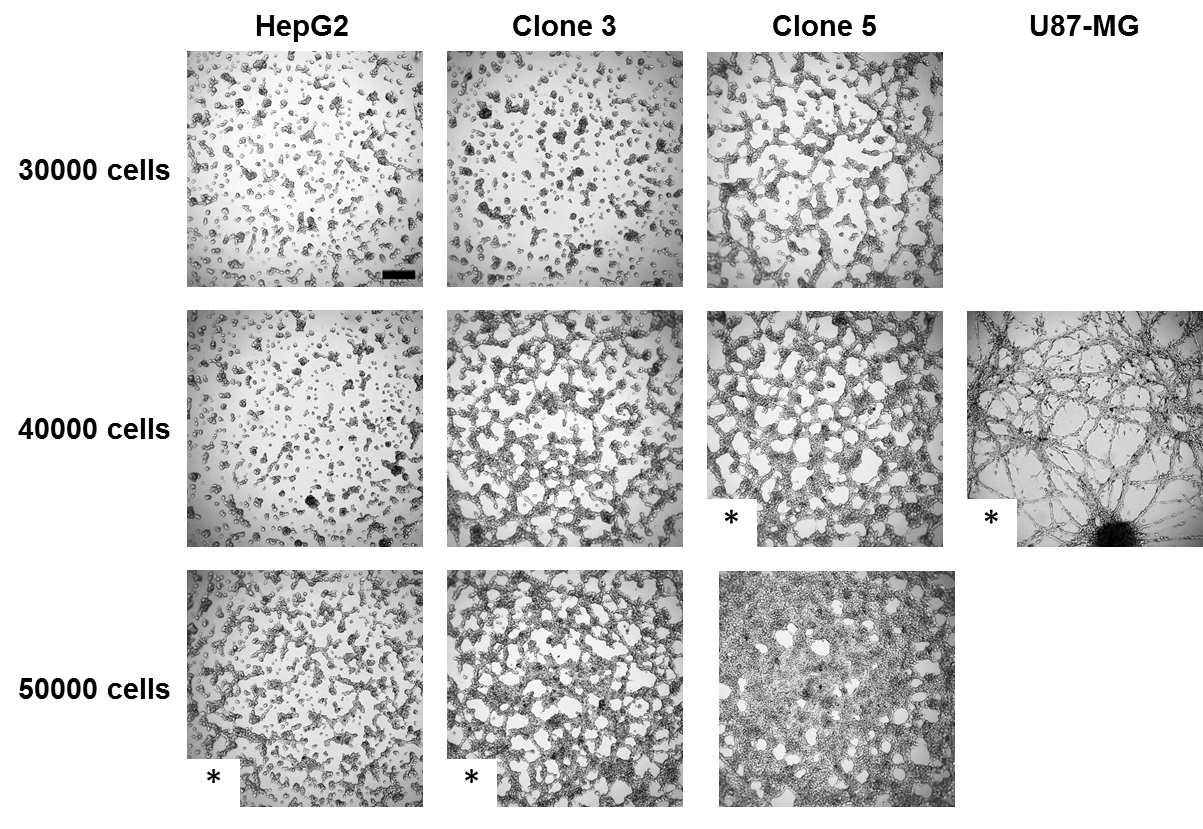


**Figure S2:** Tube Formation Assay to assess the ability of HepG2 WT, clone 3 and clone 5 cells to form vasculogenic mimicry after growth on Matrigel for 24 h. Scale – 250 µm. All images are representative for at least two independent experiments. * Images were already shown in Figure 2C.

*E-Cadherin expression is strongly reduces in clone 5 cells*


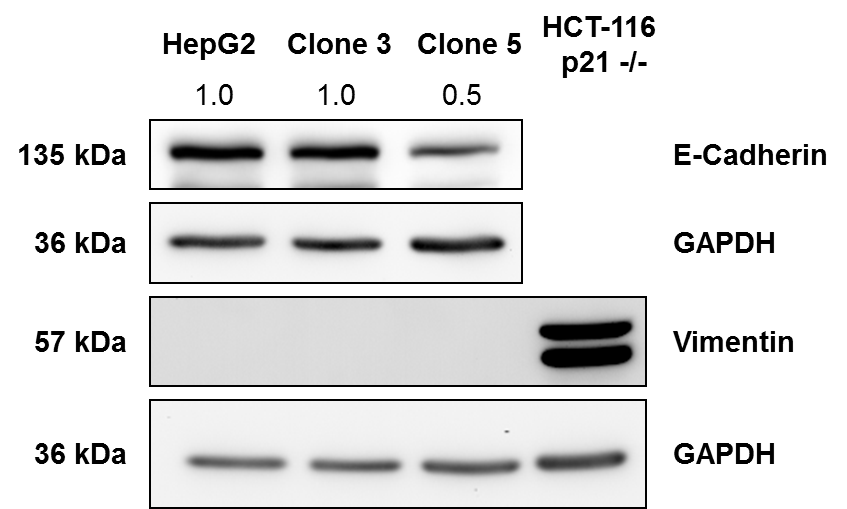


**Figure S3:** Expression of E-Cadherin and Vimentin as determined by Western Blot Analysis.
HCT-116 p21-/- cells were only used as a positive control for Vimentin and therefore not applied to the E-Cadherin Blot. The shown Western Blot is representative for two independent biological replicates.

**References**

[S1] **Castro F, Dirks WG, Fähnrich S, et al.** High-throughput SNP-based authentication of human cell lines. *Int. J. Cancer* 2013; 132; 308–14.

[S2] **Francescone III RA, Faibish M, Shao R**. A Matrigel-Based Tube Formation Assay to Assess the Vasculogenic Activity of Tumor Cells. *J. Vis. Exp.* 2011.

[S3] **El Hallani S, Boisselier B, Peglion F, et al.** A new alternative mechanism in glioblastoma vascularization: tubular vasculogenic mimicry. *Brain* 2010; 133; 973–82.

[S4] **Specht E, Kaemmerer D, Sänger J, et al.** Comparison of immunoreactive score, HER2/ *neu* score and H score for the immunohistochemical evaluation of somatostatin receptors in bronchopulmonary neuroendocrine neoplasms. *Histopathology* 2015; 67; 368–77.

[S5] **Remmele W, Stegner HE**. Recommendation for uniform definition of an immunoreactive score (IRS) for immunohistochemical estrogen receptor detection (ER-ICA) in breast cancer tissue. *Pathol.* 1987; 8; 138–40.

[S6] **Zijlstra A, Mellor R, Panzarella G, et al.** A quantitative analysis of rate-limiting steps in the metastatic cascade using human-specific real-time polymerase chain reaction. *Cancer Res.* 2002; 62; 7083–7092.
